# *rac*-Ethyl (2*Z*)-3-{2-[(*Z*)-4-eth­oxy-4-oxobut-2-en-2-yl­amino]­cyclo­hexyl­amino}­but-2-enoate

**DOI:** 10.1107/S1600536811015248

**Published:** 2011-04-29

**Authors:** Mohamed Anoir Harrad, Brahim Boualy, Mustapha Ait Ali, Larbi El Firdoussi, Corrado Rizzoli

**Affiliations:** a Equipe de Chimie de Coordination et Catalyse, Faculté des Sciences-Semlalia, BP 2390, 40001 Marrakech, Morocco; b Dipartimento di Chimica Generale ed Inorganica, Chimica Analitica, Chimica Fisica, Universitá degli Studi di Parma, Viale G. P. Usberti 17/A, I-43124 Parma, Italy

## Abstract

The asymmetric unit of the title compound, C_18_H_30_N_2_O_4_, contains two independent mol­ecules. In each mol­ecule, the cyclo­hexane ring adopts a chair conformation with equatorial orientation of the substituents, and the conformation is stabilized by two intra­molecular N—H⋯O hydrogen bonds, forming rings of *S*(6) graph-set motif. One eth­oxy group and one ethyl group are disordered over two sets of sites with refined occupancy ratios of 0.704 (2):0.296 (2) and 0.505 (3):0.495 (3), respectively. In the crystal, a weak inter­molecular C—H⋯O hydrogen inter­action is observed, involving the O atom of the major component of the disordered eth­oxy group.

## Related literature

For the synthesis and applications of β-enamino­esters, see: Spivey *et al.* (2003 ▶); Eddington *et al.* (2003 ▶); Elaridi, Thaqi *et al.* (2005 ▶); Cornils & Herrmann (1996 ▶); Venter *et al.* (2009 ▶); Elaridi, Jackson & Robinson (2005 ▶); Harrad *et al.* (2010 ▶). For related structures, see: McCann *et al.* (2001 ▶); Huang *et al.* (2008) ▶. For puckering parameters, see: Cremer & Pople (1975 ▶). For graph-set notation, see: Bernstein *et al.* (1995 ▶).
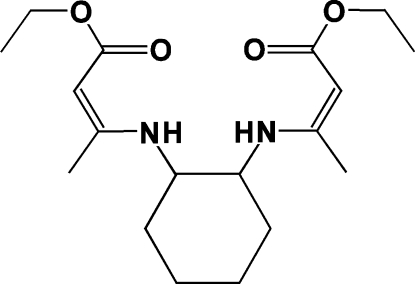


## Experimental

### 

#### Crystal data

C_18_H_30_N_2_O_4_
*M* *_r_* = 338.44
Triclinic, 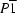

*a* = 11.1424 (9) Å
*b* = 12.7445 (6) Å
*c* = 16.3298 (11) Å
α = 90.904 (6)°
β = 109.222 (6)°
γ = 114.048 (5)°
*V* = 1969.1 (3) Å^3^
*Z* = 4
Cu *K*α radiation
μ = 0.65 mm^−1^
*T* = 294 K
0.18 × 0.15 × 0.10 mm

#### Data collection

Siemens AED diffractometer
7461 measured reflections
7189 independent reflections
5826 reflections with *I* > 2σ(*I*)
*R*_int_ = 0.006
3 standard reflections every 100 reflections  intensity decay: 0.02%

#### Refinement

*R*[*F* ^2^ > 2σ(*F* ^2^)] = 0.034
*wR*(*F* ^2^) = 0.106
*S* = 1.16
7189 reflections
467 parameters
8 restraints
H atoms treated by a mixture of independent and constrained refinement
Δρ_max_ = 0.19 e Å^−3^
Δρ_min_ = −0.14 e Å^−3^

### 

Data collection: *AED* (Belletti *et al.*, 1993 ▶); cell refinement: *AED*; data reduction: *AED*; program(s) used to solve structure: *SIR97* (Altomare *et al.*, 1999 ▶); program(s) used to refine structure: *SHELXL97* (Sheldrick, 2008 ▶); molecular graphics: *ORTEP-3 for Windows* (Farrugia, 1997 ▶) and *SCHAKAL97* (Keller, 1997 ▶); software used to prepare material for publication: *SHELXL97* and *PARST95* (Nardelli, 1995 ▶).

## Supplementary Material

Crystal structure: contains datablocks global, I. DOI: 10.1107/S1600536811015248/gk2371sup1.cif

Structure factors: contains datablocks I. DOI: 10.1107/S1600536811015248/gk2371Isup2.hkl

Supplementary material file. DOI: 10.1107/S1600536811015248/gk2371Isup3.cml

Additional supplementary materials:  crystallographic information; 3D view; checkCIF report

## Figures and Tables

**Table 1:** Hydrogen-bond geometry (Å, °)

*D*—H⋯*A*	*D*—H	H⋯*A*	*D*⋯*A*	*D*—H⋯*A*
N1—H1*N*⋯O1	0.850 (13)	2.048 (14)	2.754 (2)	140.1 (12)
N2—H2*N*⋯O3	0.865 (12)	2.009 (12)	2.720 (2)	138.7 (13)
N3—H3*N*⋯O5	0.846 (17)	2.018 (15)	2.724 (2)	140.3 (12)
N4—H4*N*⋯O7	0.811 (17)	2.112 (15)	2.754 (2)	136.1 (14)
C4—H4*A*⋯O6*A*^i^	0.97	2.48	3.400 (3)	158

Symmetry code: (i) 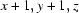.

**Fig. 1.**
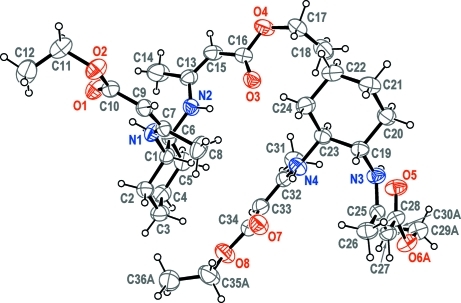
The asymmetric unit of the title compound, with displacement ellipsoids drawn at the 30% probability level. Only the major components of disorder are shown.

**Fig. 2.**
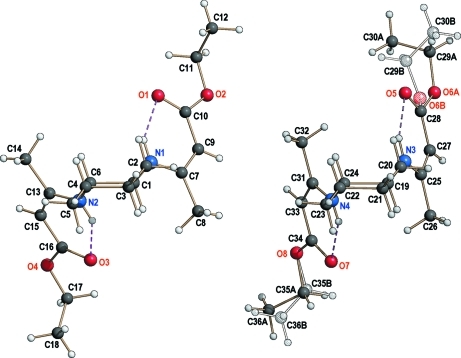
Comparison of the conformations of the two independent molecules of the title compound.

**Fig. 3.**
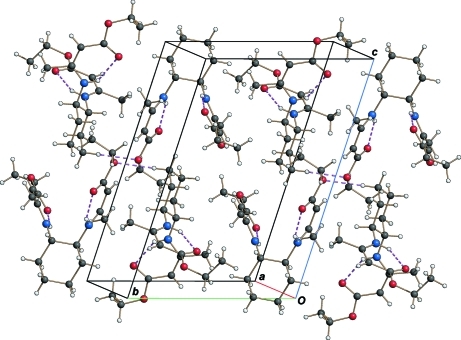
Crystal packing of the title compound with intra- and intermolecular hydrogen bonds shown as dashed lines.

## supplementary crystallographic information

### Comment

β-Enaminoesters have been extensively studied because of their applications in pharmaceutical, biochemical, biomedical and immunochemical research (Spivey *et al.*, 2003; Eddington *et al.*, 2003). These compounds are also used as precursors for the preparation of a large number of heterocyclic derivatives (Elaridi, Thaqi *et al.*, 2005) and novel organometallic complexes (Cornils & Herrmann, 1996; Venter *et al.*, 2009). Furthermore, chiral β-amino acid derivatives were prepared by enantioselective hydrogenation of β-enaminoesters (Elaridi, Jackson & Robinson, 2005). In the course of our studies in this field, we have recently reported an efficient method for the synthesis of various β-enaminoesters (Harrad *et al.*, 2010). Following our catalysis objective on the coupling of amines and keto-ester compounds, we describe herein the crystal structure of a new β-enaminoester which has been prepared using our previously mentioned method by the dicondensation of trans-cyclohexane-1,2-diamine with 3-oxo-butyric acid ethyl ester under solvent-free conditions.

The asymmetric unit of the title compound (Fig. 1) consists of two independent molecules differing mainly in the orientation of the aminobutenoate groups (Fig. 2), as indicated by the dihedral angles of 82.23 (3) and 51.57 (3)° between the mean planes through N1/O1/O2/C8—C10 and N2/O3/O4/C13—C16 in one molecule, and through N3/O5/O6A/O6B/C25—C28 and N4/O7/O8/C31—C24 in the other molecule. The molecular conformations, where the substituents are equatorially oriented with respect to the cyclohexane rings, are similar to those observed for the related compound *trans*-*N*,*N*'-bis(4-oxopent-2-en-2-yl)-1,2-diaminocyclohexane (McCann *et al.*, 2001; Huang *et al.*, 2009). In each aminobutenoate group an intramolecular N—H···O hydrogen bond (Table 1) is present, forming a ring of *S*(6) graph-set motif (Bernstein *et al.*, 1995). The cyclohexane rings adopt a chair conformations with puckering parameters *Q*, θ and φ (Cremer & Pople, 1975) of 0.5691 (11) Å, 179.21 (19)°, -105 (8)° and 0.5723 (15) Å, 178.86 (15)°, -67 (8)° for rings C1–C6 and C19–C24, respectively. In one molecule, the O6/C29–C30 ethoxy group and the C35–C36 ethyl group are disordered over two orientations with refined site occupancy ratios of 0.704 (2):0.296 (2) and 0.505 (3):0.495 (3), respectively. In the crystal structure (Fig. 3), one weak C—H···O hydrogen bond (Table 1) is observed, involving the oxygen atom of the major component of the disordered ethoxy group as acceptor.

### Experimental

To a stirred mixture of 3-oxo-butyric acid ethyl ester (1.7 mmol), *trans*-cyclohexane-1,2-diamine (0.85 mmol) and Ca(CF_3_COO)_2_ (0.17 mmol) at room temperature 10 ml of distilled water was added, and the residue extracted with diethyl ether (3 × 25 ml). The organic layer was dried over Na_2_SO_4_ and the solvent removed under reduced pressure. The title β-enaminoester was obtained by column chromatography over silica gel using a mixture of *n*-hexane/ethyl acetate (5:95 *v*/*v*) as eluent (yield 90%; m. p. 160 °C). Crystals suitable for X-ray analysis were obtained on slow evaporation of the solvent at room temperature.

### Refinement

The amine H atoms were located in a difference Fourier map and refined freely. All other H atoms were placed at calculated positions and refined using a riding model approximation, with C—H = 0.93–0.98 Å, and with *U*_iso_(H) = 1.2 *U*_eq_(C) or 1.5 *U*_eq_(C) for methyl H atoms. One ethoxy group (O6/C29–C30) and one ethyl group (C35–C36) are disordered over two orientations (called A and B) with refined site occupancy ratios of 0.704 (2):0.296 (2) and 0.505 (3):0.495 (3), respectively. During the refinement, the O—C and C—C bond distances within the disordered groups were restrained to be 1.45 (1) and 1.49 (1) Å, respectively, and the anisotropic displacement parameters of the pairs of the disordered atoms were set equal by the command EADP (Sheldrick, 2008).

### Crystal data

**Table:**

C_18_H_30_N_2_O_4_	*Z* = 4
*M**_r_* = 338.44	*F*(000) = 736
Triclinic, *P*1	*D*_x_ = 1.142 Mg m^−^^3^
Hall symbol: -P 1	Cu *K*α radiation, λ = 1.54178 Å
*a* = 11.1424 (9) Å	Cell parameters from 48 reflections
*b* = 12.7445 (6) Å	θ = 18.4–29.8°
*c* = 16.3298 (11) Å	µ = 0.65 mm^−^^1^
α = 90.904 (6)°	*T* = 294 K
β = 109.222 (6)°	Irregular block, colourless
γ = 114.048 (5)°	0.18 × 0.15 × 0.10 mm
*V* = 1969.1 (3) Å^3^	

### Data collection

**Table:**

Siemens AED diffractometer	*R*_int_ = 0.006
Radiation source: fine-focus sealed tube	θ_max_ = 68.0°, θ_min_ = 2.9°
graphite	*h* = −13→12
θ/2θ scans	*k* = −15→9
7461 measured reflections	*l* = −18→19
7189 independent reflections	3 standard reflections every 100 reflections
5826 reflections with *I* > 2σ(*I*)	intensity decay: 0.02%

### Refinement

**Table:**

Refinement on *F*^2^	Secondary atom site location: difference Fourier map
Least-squares matrix: full	Hydrogen site location: inferred from neighbouring sites
*R*[*F*^2^ > 2σ(*F*^2^)] = 0.034	H atoms treated by a mixture of independent and constrained refinement
*wR*(*F*^2^) = 0.106	*w* = 1/[σ^2^(*F*_o_^2^) + (0.0604*P*)^2^ + 0.0296*P*] where *P* = (*F*_o_^2^ + 2*F*_c_^2^)/3
*S* = 1.16	(Δ/σ)_max_ < 0.001
7189 reflections	Δρ_max_ = 0.19 e Å^−^^3^
467 parameters	Δρ_min_ = −0.14 e Å^−^^3^
8 restraints	Extinction correction: *SHELXL97* (Sheldrick, 2008)
Primary atom site location: structure-invariant direct methods	Extinction coefficient: 0.0100 (5)

### Special details

**Table:**

Refinement. Refinement of *F*^2^ against ALL reflections. The weighted *R*-factor *wR* and goodness of fit *S* are based on *F*^2^, conventional *R*-factors *R* are based on *F*, with *F* set to zero for negative *F*^2^. The threshold expression of *F*^2^ > σ(*F*^2^) is used only for calculating *R*-factors(gt) *etc*. and is not relevant to the choice of reflections for refinement. *R*-factors based on *F*^2^ are statistically about twice as large as those based on *F*, and *R*- factors based on ALL data will be even larger.

### Fractional atomic coordinates and isotropic or equivalent isotropic displacement parameters (Å^2^)

**Table:**

	*x*	*y*	*z*	*U*_iso_*/*U*_eq_	Occ. (<1)
O1	0.65218 (9)	0.84797 (6)	0.08853 (5)	0.0814 (2)	
O2	0.58185 (10)	0.74147 (7)	−0.04449 (5)	0.0864 (2)	
O3	0.11165 (8)	0.64888 (7)	0.18740 (5)	0.0774 (2)	
O4	−0.03250 (9)	0.73472 (8)	0.13437 (6)	0.0912 (3)	
O5	−0.19007 (10)	0.18776 (8)	0.38766 (5)	0.0895 (3)	
O6A	−0.1211 (2)	0.10330 (16)	0.50344 (14)	0.0829 (5)	0.704 (2)
O6B	−0.0647 (6)	0.1425 (4)	0.5121 (4)	0.0829 (5)	0.296 (2)
O7	0.34330 (8)	0.27768 (7)	0.26921 (6)	0.0846 (2)	
O8	0.52581 (9)	0.38869 (8)	0.39309 (7)	0.0963 (3)	
N1	0.55037 (10)	0.72198 (8)	0.20595 (6)	0.0684 (2)	
H1N	0.5994 (13)	0.7854 (12)	0.1927 (8)	0.082 (4)*	
N2	0.38841 (10)	0.79819 (8)	0.27442 (7)	0.0727 (2)	
H2N	0.3211 (14)	0.7281 (12)	0.2553 (8)	0.084 (4)*	
N3	−0.13927 (10)	0.13606 (8)	0.24363 (6)	0.0687 (2)	
H3N	−0.1697 (13)	0.1707 (11)	0.2705 (8)	0.080 (4)*	
N4	0.11045 (10)	0.32223 (9)	0.23049 (6)	0.0719 (2)	
H4N	0.1472 (13)	0.2852 (11)	0.2165 (8)	0.082 (4)*	
C1	0.55684 (11)	0.71559 (9)	0.29601 (6)	0.0635 (2)	
H1	0.4829	0.6400	0.2960	0.076*	
C2	0.69934 (12)	0.72291 (10)	0.35581 (8)	0.0756 (3)	
H2A	0.7131	0.6592	0.3338	0.091*	
H2B	0.7744	0.7955	0.3542	0.091*	
C3	0.70863 (14)	0.71697 (11)	0.45078 (8)	0.0846 (3)	
H3A	0.6395	0.6415	0.4536	0.102*	
H3B	0.8018	0.7256	0.4871	0.102*	
C4	0.68137 (14)	0.81250 (11)	0.48572 (8)	0.0863 (3)	
H4A	0.7565	0.8879	0.4890	0.104*	
H4B	0.6816	0.8045	0.5447	0.104*	
C5	0.53939 (13)	0.80654 (11)	0.42641 (8)	0.0819 (3)	
H5A	0.4639	0.7346	0.4285	0.098*	
H5B	0.5272	0.8710	0.4487	0.098*	
C6	0.52777 (11)	0.81168 (9)	0.33084 (7)	0.0664 (2)	
H6	0.5987	0.8876	0.3285	0.080*	
C7	0.48965 (10)	0.63196 (8)	0.13905 (6)	0.0608 (2)	
C8	0.40309 (15)	0.51312 (10)	0.15353 (9)	0.0937 (4)	
H8A	0.4628	0.4896	0.1987	0.141*	
H8B	0.3609	0.4582	0.0998	0.141*	
H8C	0.3302	0.5155	0.1714	0.141*	
C9	0.50447 (10)	0.64528 (9)	0.05978 (7)	0.0648 (2)	
H9	0.4586	0.5793	0.0162	0.078*	
C10	0.58526 (11)	0.75309 (9)	0.03918 (7)	0.0655 (2)	
C11	0.65923 (17)	0.84598 (13)	−0.07360 (9)	0.0971 (4)	
H11A	0.6510	0.9113	−0.0488	0.117*	
H11B	0.6176	0.8358	−0.1372	0.117*	
C12	0.80899 (19)	0.87259 (17)	−0.04739 (13)	0.1255 (6)	
H12A	0.8566	0.9426	−0.0674	0.188*	
H12B	0.8177	0.8091	−0.0732	0.188*	
H12C	0.8508	0.8836	0.0156	0.188*	
C13	0.34359 (13)	0.88097 (10)	0.25392 (8)	0.0779 (3)	
C14	0.45322 (18)	1.00673 (12)	0.28361 (13)	0.1315 (7)	
H14A	0.4072	1.0572	0.2707	0.197*	
H14B	0.5175	1.0224	0.2531	0.197*	
H14C	0.5046	1.0204	0.3459	0.197*	
C15	0.20513 (13)	0.85458 (10)	0.20757 (7)	0.0795 (3)	
H15	0.1805	0.9157	0.1951	0.095*	
C16	0.09706 (12)	0.73879 (10)	0.17749 (7)	0.0699 (3)	
C17	−0.15032 (13)	0.61954 (12)	0.10095 (8)	0.0878 (4)	
H17A	−0.1213	0.5689	0.0757	0.105*	
H17B	−0.2272	0.6252	0.0544	0.105*	
C18	−0.20206 (15)	0.56635 (16)	0.17051 (10)	0.1087 (5)	
H18A	−0.2798	0.4907	0.1452	0.163*	
H18B	−0.2327	0.6153	0.1950	0.163*	
H18C	−0.1270	0.5587	0.2161	0.163*	
C19	−0.13341 (11)	0.16744 (9)	0.15936 (7)	0.0646 (2)	
H19	−0.0979	0.1202	0.1354	0.077*	
C20	−0.28197 (12)	0.14077 (10)	0.09474 (7)	0.0760 (3)	
H20A	−0.3426	0.0579	0.0856	0.091*	
H20B	−0.3214	0.1824	0.1197	0.091*	
C21	−0.27846 (14)	0.17667 (11)	0.00643 (8)	0.0837 (3)	
H21A	−0.2467	0.1302	−0.0209	0.100*	
H21B	−0.3730	0.1616	−0.0324	0.100*	
C22	−0.18062 (14)	0.30461 (12)	0.01838 (8)	0.0833 (3)	
H22A	−0.2169	0.3513	0.0410	0.100*	
H22B	−0.1776	0.3245	−0.0382	0.100*	
C23	−0.03151 (13)	0.33309 (12)	0.08190 (8)	0.0817 (3)	
H23A	0.0276	0.4162	0.0908	0.098*	
H23B	0.0085	0.2926	0.0565	0.098*	
C24	−0.03252 (11)	0.29689 (9)	0.17064 (7)	0.0652 (2)	
H24	−0.0665	0.3432	0.1971	0.078*	
C25	−0.07559 (10)	0.07824 (8)	0.29520 (7)	0.0607 (2)	
C26	−0.00907 (14)	0.01806 (11)	0.25829 (8)	0.0788 (3)	
H26A	0.0647	0.0746	0.2427	0.118*	
H26B	−0.0793	−0.0381	0.2069	0.118*	
H26C	0.0300	−0.0209	0.3017	0.118*	
C27	−0.07262 (11)	0.07126 (9)	0.37959 (7)	0.0654 (2)	
H27	−0.0317	0.0267	0.4114	0.078*	
C28	−0.12888 (13)	0.12870 (9)	0.42072 (7)	0.0725 (3)	
C29A	−0.1870 (3)	0.14951 (19)	0.54811 (14)	0.0902 (5)	0.704 (2)
H29A	−0.2160	0.0993	0.5888	0.108*	0.704 (2)
H29B	−0.2707	0.1505	0.5052	0.108*	0.704 (2)
C30A	−0.0877 (3)	0.2695 (2)	0.59710 (16)	0.1157 (7)	0.704 (2)
H30A	−0.1333	0.2985	0.6259	0.174*	0.704 (2)
H30B	−0.0598	0.3194	0.5568	0.174*	0.704 (2)
H30C	−0.0056	0.2683	0.6403	0.174*	0.704 (2)
C29B	−0.0883 (6)	0.2124 (5)	0.5686 (3)	0.0902 (5)	0.296 (2)
H29C	−0.0699	0.2880	0.5509	0.108*	0.296 (2)
H29D	−0.0242	0.2246	0.6288	0.108*	0.296 (2)
C30B	−0.2357 (7)	0.1538 (6)	0.5640 (4)	0.1157 (7)	0.296 (2)
H30D	−0.2509	0.2013	0.6020	0.174*	0.296 (2)
H30E	−0.2532	0.0796	0.5824	0.174*	0.296 (2)
H30F	−0.2988	0.1424	0.5045	0.174*	0.296 (2)
C31	0.18551 (11)	0.39366 (9)	0.30854 (7)	0.0653 (2)	
C32	0.11850 (15)	0.45730 (13)	0.34209 (8)	0.0911 (4)	
H32A	0.0876	0.5000	0.2987	0.137*	
H32B	0.1863	0.5105	0.3955	0.137*	
H32C	0.0389	0.4020	0.3535	0.137*	
C33	0.32099 (11)	0.41186 (9)	0.35799 (7)	0.0692 (3)	
H33	0.3689	0.4659	0.4101	0.083*	
C34	0.39224 (11)	0.35293 (9)	0.33421 (8)	0.0701 (3)	
C35A	0.6064 (10)	0.3370 (8)	0.3684 (8)	0.1107 (14)	0.505 (3)
H35A	0.5625	0.3052	0.3059	0.133*	0.505 (3)
H35B	0.6079	0.2739	0.4005	0.133*	0.505 (3)
C36A	0.7530 (4)	0.4278 (3)	0.3891 (3)	0.1138 (9)	0.505 (3)
H36A	0.8069	0.3938	0.3736	0.171*	0.505 (3)
H36B	0.7958	0.4591	0.4509	0.171*	0.505 (3)
H36C	0.7510	0.4891	0.3561	0.171*	0.505 (3)
C35B	0.6115 (10)	0.3321 (9)	0.3864 (8)	0.1107 (14)	0.495 (3)
H35C	0.5535	0.2491	0.3655	0.133*	0.495 (3)
H35D	0.6832	0.3434	0.4435	0.133*	0.495 (3)
C36B	0.6779 (4)	0.3848 (3)	0.3239 (3)	0.1138 (9)	0.495 (3)
H36D	0.7310	0.3454	0.3148	0.171*	0.495 (3)
H36E	0.7401	0.4658	0.3472	0.171*	0.495 (3)
H36F	0.6062	0.3777	0.2688	0.171*	0.495 (3)

### Atomic displacement parameters (Å^2^)

**Table:**

	*U*^11^	*U*^22^	*U*^33^	*U*^12^	*U*^13^	*U*^23^
O1	0.0996 (6)	0.0589 (4)	0.0826 (5)	0.0259 (4)	0.0411 (4)	0.0138 (4)
O2	0.1099 (6)	0.0779 (5)	0.0716 (5)	0.0353 (5)	0.0407 (4)	0.0190 (4)
O3	0.0683 (4)	0.0726 (5)	0.0910 (5)	0.0352 (4)	0.0234 (4)	0.0129 (4)
O4	0.0796 (5)	0.0919 (6)	0.0928 (6)	0.0482 (5)	0.0072 (4)	0.0111 (4)
O5	0.1190 (7)	0.1038 (6)	0.0856 (5)	0.0769 (6)	0.0486 (5)	0.0328 (5)
O6A	0.1189 (17)	0.0739 (12)	0.0744 (7)	0.0502 (11)	0.0471 (12)	0.0226 (9)
O6B	0.1189 (17)	0.0739 (12)	0.0744 (7)	0.0502 (11)	0.0471 (12)	0.0226 (9)
O7	0.0722 (5)	0.0796 (5)	0.0976 (6)	0.0342 (4)	0.0253 (4)	0.0048 (4)
O8	0.0653 (5)	0.0818 (5)	0.1212 (7)	0.0336 (4)	0.0085 (4)	−0.0023 (5)
N1	0.0743 (5)	0.0544 (5)	0.0685 (5)	0.0177 (4)	0.0301 (4)	0.0123 (4)
N2	0.0631 (5)	0.0594 (5)	0.0851 (6)	0.0232 (4)	0.0194 (4)	0.0076 (4)
N3	0.0763 (6)	0.0798 (6)	0.0707 (5)	0.0477 (5)	0.0340 (4)	0.0227 (4)
N4	0.0635 (5)	0.0806 (6)	0.0752 (6)	0.0370 (5)	0.0231 (4)	0.0075 (5)
C1	0.0623 (5)	0.0550 (5)	0.0642 (5)	0.0178 (4)	0.0227 (4)	0.0114 (4)
C2	0.0709 (6)	0.0699 (6)	0.0818 (7)	0.0314 (5)	0.0220 (5)	0.0158 (5)
C3	0.0831 (8)	0.0752 (7)	0.0766 (7)	0.0302 (6)	0.0122 (6)	0.0179 (6)
C4	0.0909 (8)	0.0780 (7)	0.0644 (6)	0.0235 (6)	0.0152 (6)	0.0080 (5)
C5	0.0844 (8)	0.0805 (7)	0.0737 (7)	0.0289 (6)	0.0302 (6)	0.0055 (5)
C6	0.0609 (5)	0.0579 (5)	0.0688 (6)	0.0187 (4)	0.0194 (5)	0.0080 (4)
C7	0.0515 (5)	0.0560 (5)	0.0645 (5)	0.0194 (4)	0.0144 (4)	0.0089 (4)
C8	0.1030 (9)	0.0618 (7)	0.0816 (8)	0.0023 (6)	0.0360 (7)	0.0035 (5)
C9	0.0624 (6)	0.0586 (5)	0.0640 (6)	0.0223 (4)	0.0173 (4)	0.0070 (4)
C10	0.0709 (6)	0.0646 (6)	0.0658 (6)	0.0342 (5)	0.0248 (5)	0.0148 (5)
C11	0.1316 (12)	0.0907 (9)	0.0856 (8)	0.0509 (9)	0.0557 (8)	0.0361 (7)
C12	0.1146 (13)	0.1228 (13)	0.1317 (14)	0.0328 (10)	0.0597 (11)	0.0457 (11)
C13	0.0854 (7)	0.0616 (6)	0.0734 (7)	0.0286 (5)	0.0176 (6)	0.0133 (5)
C14	0.1176 (12)	0.0606 (8)	0.1555 (15)	0.0270 (8)	−0.0074 (11)	0.0155 (8)
C15	0.0893 (8)	0.0714 (7)	0.0753 (7)	0.0441 (6)	0.0156 (6)	0.0138 (5)
C16	0.0733 (6)	0.0781 (7)	0.0618 (6)	0.0411 (6)	0.0186 (5)	0.0114 (5)
C17	0.0700 (7)	0.1016 (9)	0.0771 (7)	0.0386 (7)	0.0084 (6)	0.0057 (6)
C18	0.0777 (8)	0.1517 (14)	0.0942 (9)	0.0514 (9)	0.0268 (7)	0.0191 (9)
C19	0.0687 (6)	0.0691 (6)	0.0656 (6)	0.0388 (5)	0.0250 (5)	0.0139 (5)
C20	0.0694 (6)	0.0742 (7)	0.0768 (7)	0.0324 (5)	0.0167 (5)	0.0085 (5)
C21	0.0901 (8)	0.0898 (8)	0.0687 (6)	0.0505 (7)	0.0123 (6)	0.0086 (6)
C22	0.0974 (8)	0.0956 (8)	0.0738 (7)	0.0589 (7)	0.0297 (6)	0.0291 (6)
C23	0.0850 (8)	0.0902 (8)	0.0843 (7)	0.0467 (7)	0.0369 (6)	0.0329 (6)
C24	0.0631 (6)	0.0708 (6)	0.0684 (6)	0.0358 (5)	0.0237 (5)	0.0129 (5)
C25	0.0551 (5)	0.0535 (5)	0.0707 (6)	0.0238 (4)	0.0196 (4)	0.0098 (4)
C26	0.0933 (8)	0.0823 (7)	0.0818 (7)	0.0561 (7)	0.0344 (6)	0.0194 (6)
C27	0.0709 (6)	0.0588 (5)	0.0686 (6)	0.0329 (5)	0.0223 (5)	0.0141 (4)
C28	0.0880 (7)	0.0653 (6)	0.0714 (6)	0.0385 (6)	0.0314 (5)	0.0177 (5)
C29A	0.1123 (16)	0.0960 (15)	0.0813 (11)	0.0553 (12)	0.0459 (12)	0.0170 (10)
C30A	0.1343 (18)	0.1182 (16)	0.1043 (15)	0.0694 (14)	0.0388 (13)	−0.0099 (12)
C29B	0.1123 (16)	0.0960 (15)	0.0813 (11)	0.0553 (12)	0.0459 (12)	0.0170 (10)
C30B	0.1343 (18)	0.1182 (16)	0.1043 (15)	0.0694 (14)	0.0388 (13)	−0.0099 (12)
C31	0.0716 (6)	0.0657 (6)	0.0641 (6)	0.0310 (5)	0.0297 (5)	0.0196 (5)
C32	0.0950 (9)	0.1066 (10)	0.0821 (8)	0.0563 (8)	0.0301 (7)	0.0042 (7)
C33	0.0715 (6)	0.0686 (6)	0.0658 (6)	0.0298 (5)	0.0238 (5)	0.0167 (5)
C34	0.0623 (6)	0.0603 (6)	0.0805 (7)	0.0228 (5)	0.0225 (5)	0.0189 (5)
C35A	0.0754 (10)	0.0835 (10)	0.157 (4)	0.0428 (8)	0.0135 (18)	0.0046 (19)
C36A	0.102 (2)	0.0986 (19)	0.163 (3)	0.0561 (17)	0.0601 (18)	0.0378 (19)
C35B	0.0754 (10)	0.0835 (10)	0.157 (4)	0.0428 (8)	0.0135 (18)	0.0046 (19)
C36B	0.102 (2)	0.0986 (19)	0.163 (3)	0.0561 (17)	0.0601 (18)	0.0378 (19)

### Geometric parameters (Å, °)

**Table:**

O1—C10	1.2273 (12)	C15—H15	0.9300
O2—C10	1.3584 (13)	C17—C18	1.490 (2)
O2—C11	1.4482 (15)	C17—H17A	0.9700
O3—C16	1.2272 (13)	C17—H17B	0.9700
O4—C16	1.3605 (13)	C18—H18A	0.9600
O4—C17	1.4520 (16)	C18—H18B	0.9600
O5—C28	1.2294 (13)	C18—H18C	0.9600
O6A—C28	1.377 (2)	C19—C20	1.5327 (14)
O6A—C29A	1.453 (3)	C19—C24	1.5368 (15)
O6B—C28	1.398 (6)	C19—H19	0.9800
O6B—C29B	1.429 (6)	C20—C21	1.5277 (16)
O7—C34	1.2264 (14)	C20—H20A	0.9700
O8—C34	1.3558 (13)	C20—H20B	0.9700
O8—C35B	1.439 (7)	C21—C22	1.5121 (19)
O8—C35A	1.455 (7)	C21—H21A	0.9700
N1—C7	1.3448 (13)	C21—H21B	0.9700
N1—C1	1.4538 (13)	C22—C23	1.5264 (17)
N1—H1N	0.850 (13)	C22—H22A	0.9700
N2—C13	1.3435 (14)	C22—H22B	0.9700
N2—C6	1.4577 (13)	C23—C24	1.5298 (14)
N2—H2N	0.865 (13)	C23—H23A	0.9700
N3—C25	1.3446 (13)	C23—H23B	0.9700
N3—C19	1.4537 (13)	C24—H24	0.9800
N3—H3N	0.847 (13)	C25—C27	1.3727 (14)
N4—C31	1.3392 (14)	C25—C26	1.4979 (14)
N4—C24	1.4639 (13)	C26—H26A	0.9600
N4—H4N	0.811 (13)	C26—H26B	0.9600
C1—C2	1.5293 (14)	C26—H26C	0.9600
C1—C6	1.5332 (15)	C27—C28	1.4197 (15)
C1—H1	0.9800	C27—H27	0.9300
C2—C3	1.5251 (16)	C29A—C30A	1.484 (3)
C2—H2A	0.9700	C29A—H29A	0.9700
C2—H2B	0.9700	C29A—H29B	0.9700
C3—C4	1.5145 (18)	C30A—H30A	0.9600
C3—H3A	0.9700	C30A—H30B	0.9600
C3—H3B	0.9700	C30A—H30C	0.9600
C4—C5	1.5288 (18)	C29B—C30B	1.475 (7)
C4—H4A	0.9700	C29B—H29C	0.9700
C4—H4B	0.9700	C29B—H29D	0.9700
C5—C6	1.5277 (15)	C30B—H30D	0.9600
C5—H5A	0.9700	C30B—H30E	0.9600
C5—H5B	0.9700	C30B—H30F	0.9600
C6—H6	0.9800	C31—C33	1.3751 (15)
C7—C9	1.3626 (14)	C31—C32	1.5048 (15)
C7—C8	1.5003 (15)	C32—H32A	0.9600
C8—H8A	0.9600	C32—H32B	0.9600
C8—H8B	0.9600	C32—H32C	0.9600
C8—H8C	0.9600	C33—C34	1.4231 (15)
C9—C10	1.4202 (14)	C33—H33	0.9300
C9—H9	0.9300	C35A—C36A	1.485 (9)
C11—C12	1.465 (2)	C35A—H35A	0.9700
C11—H11A	0.9700	C35A—H35B	0.9700
C11—H11B	0.9700	C36A—H36A	0.9600
C12—H12A	0.9600	C36A—H36B	0.9600
C12—H12B	0.9600	C36A—H36C	0.9600
C12—H12C	0.9600	C35B—C36B	1.468 (9)
C13—C15	1.3662 (17)	C35B—H35C	0.9700
C13—C14	1.5119 (18)	C35B—H35D	0.9700
C14—H14A	0.9600	C36B—H36D	0.9600
C14—H14B	0.9600	C36B—H36E	0.9600
C14—H14C	0.9600	C36B—H36F	0.9600
C15—C16	1.4172 (16)		
			
C10—O2—C11	117.28 (10)	C20—C19—H19	108.5
C16—O4—C17	116.58 (9)	C24—C19—H19	108.5
C28—O6A—C29A	117.05 (18)	C21—C20—C19	111.11 (10)
C28—O6B—C29B	119.8 (5)	C21—C20—H20A	109.4
C34—O8—C35B	121.2 (5)	C19—C20—H20A	109.4
C34—O8—C35A	114.2 (5)	C21—C20—H20B	109.4
C7—N1—C1	126.83 (9)	C19—C20—H20B	109.4
C7—N1—H1N	111.8 (8)	H20A—C20—H20B	108.0
C1—N1—H1N	120.6 (8)	C22—C21—C20	111.03 (10)
C13—N2—C6	128.79 (10)	C22—C21—H21A	109.4
C13—N2—H2N	113.1 (8)	C20—C21—H21A	109.4
C6—N2—H2N	117.7 (8)	C22—C21—H21B	109.4
C25—N3—C19	128.67 (9)	C20—C21—H21B	109.4
C25—N3—H3N	112.5 (8)	H21A—C21—H21B	108.0
C19—N3—H3N	117.2 (8)	C21—C22—C23	111.17 (10)
C31—N4—C24	128.22 (9)	C21—C22—H22A	109.4
C31—N4—H4N	115.3 (9)	C23—C22—H22A	109.4
C24—N4—H4N	116.3 (9)	C21—C22—H22B	109.4
N1—C1—C2	111.09 (9)	C23—C22—H22B	109.4
N1—C1—C6	110.54 (8)	H22A—C22—H22B	108.0
C2—C1—C6	110.93 (8)	C22—C23—C24	111.12 (9)
N1—C1—H1	108.0	C22—C23—H23A	109.4
C2—C1—H1	108.0	C24—C23—H23A	109.4
C6—C1—H1	108.0	C22—C23—H23B	109.4
C3—C2—C1	111.95 (9)	C24—C23—H23B	109.4
C3—C2—H2A	109.2	H23A—C23—H23B	108.0
C1—C2—H2A	109.2	N4—C24—C23	110.76 (9)
C3—C2—H2B	109.2	N4—C24—C19	110.83 (8)
C1—C2—H2B	109.2	C23—C24—C19	111.46 (9)
H2A—C2—H2B	107.9	N4—C24—H24	107.9
C4—C3—C2	110.45 (10)	C23—C24—H24	107.9
C4—C3—H3A	109.6	C19—C24—H24	107.9
C2—C3—H3A	109.6	N3—C25—C27	121.75 (9)
C4—C3—H3B	109.6	N3—C25—C26	118.67 (9)
C2—C3—H3B	109.6	C27—C25—C26	119.57 (9)
H3A—C3—H3B	108.1	C25—C26—H26A	109.5
C3—C4—C5	111.02 (10)	C25—C26—H26B	109.5
C3—C4—H4A	109.4	H26A—C26—H26B	109.5
C5—C4—H4A	109.4	C25—C26—H26C	109.5
C3—C4—H4B	109.4	H26A—C26—H26C	109.5
C5—C4—H4B	109.4	H26B—C26—H26C	109.5
H4A—C4—H4B	108.0	C25—C27—C28	123.28 (9)
C6—C5—C4	112.53 (10)	C25—C27—H27	118.4
C6—C5—H5A	109.1	C28—C27—H27	118.4
C4—C5—H5A	109.1	O5—C28—O6A	120.10 (13)
C6—C5—H5B	109.1	O5—C28—O6B	121.0 (2)
C4—C5—H5B	109.1	O5—C28—C27	126.94 (10)
H5A—C5—H5B	107.8	O6A—C28—C27	112.70 (12)
N2—C6—C5	111.60 (9)	O6B—C28—C27	108.9 (3)
N2—C6—C1	109.13 (8)	O6A—C29A—C30A	110.8 (2)
C5—C6—C1	110.09 (9)	O6A—C29A—H29A	109.5
N2—C6—H6	108.7	C30A—C29A—H29A	109.5
C5—C6—H6	108.7	O6A—C29A—H29B	109.5
C1—C6—H6	108.7	C30A—C29A—H29B	109.5
N1—C7—C9	122.42 (9)	H29A—C29A—H29B	108.1
N1—C7—C8	118.00 (10)	C29A—C30A—H30A	109.5
C9—C7—C8	119.59 (9)	C29A—C30A—H30B	109.5
C7—C8—H8A	109.5	H30A—C30A—H30B	109.5
C7—C8—H8B	109.5	C29A—C30A—H30C	109.5
H8A—C8—H8B	109.5	H30A—C30A—H30C	109.5
C7—C8—H8C	109.5	H30B—C30A—H30C	109.5
H8A—C8—H8C	109.5	O6B—C29B—C30B	109.9 (5)
H8B—C8—H8C	109.5	O6B—C29B—H29C	109.7
C7—C9—C10	124.40 (9)	C30B—C29B—H29C	109.7
C7—C9—H9	117.8	O6B—C29B—H29D	109.7
C10—C9—H9	117.8	C30B—C29B—H29D	109.7
O1—C10—O2	121.51 (10)	H29C—C29B—H29D	108.2
O1—C10—C9	126.35 (10)	C29B—C30B—H30D	109.5
O2—C10—C9	112.14 (9)	C29B—C30B—H30E	109.5
O2—C11—C12	112.02 (12)	H30D—C30B—H30E	109.5
O2—C11—H11A	109.2	C29B—C30B—H30F	109.5
C12—C11—H11A	109.2	H30D—C30B—H30F	109.5
O2—C11—H11B	109.2	H30E—C30B—H30F	109.5
C12—C11—H11B	109.2	N4—C31—C33	122.59 (10)
H11A—C11—H11B	107.9	N4—C31—C32	118.16 (10)
C11—C12—H12A	109.5	C33—C31—C32	119.25 (10)
C11—C12—H12B	109.5	C31—C32—H32A	109.5
H12A—C12—H12B	109.5	C31—C32—H32B	109.5
C11—C12—H12C	109.5	H32A—C32—H32B	109.5
H12A—C12—H12C	109.5	C31—C32—H32C	109.5
H12B—C12—H12C	109.5	H32A—C32—H32C	109.5
N2—C13—C15	122.28 (11)	H32B—C32—H32C	109.5
N2—C13—C14	117.39 (11)	C31—C33—C34	123.87 (10)
C15—C13—C14	120.33 (11)	C31—C33—H33	118.1
C13—C14—H14A	109.5	C34—C33—H33	118.1
C13—C14—H14B	109.5	O7—C34—O8	121.61 (10)
H14A—C14—H14B	109.5	O7—C34—C33	126.26 (10)
C13—C14—H14C	109.5	O8—C34—C33	112.13 (10)
H14A—C14—H14C	109.5	O8—C35A—C36A	109.2 (6)
H14B—C14—H14C	109.5	O8—C35A—H35A	109.8
C13—C15—C16	123.31 (10)	C36A—C35A—H35A	109.8
C13—C15—H15	118.3	O8—C35A—H35B	109.8
C16—C15—H15	118.3	C36A—C35A—H35B	109.8
O3—C16—O4	120.92 (10)	H35A—C35A—H35B	108.3
O3—C16—C15	126.59 (10)	C35A—C36A—H36A	109.5
O4—C16—C15	112.49 (10)	C35A—C36A—H36B	109.5
O4—C17—C18	112.64 (11)	H36A—C36A—H36B	109.5
O4—C17—H17A	109.1	C35A—C36A—H36C	109.5
C18—C17—H17A	109.1	H36A—C36A—H36C	109.5
O4—C17—H17B	109.1	H36B—C36A—H36C	109.5
C18—C17—H17B	109.1	O8—C35B—C36B	107.3 (7)
H17A—C17—H17B	107.8	O8—C35B—H35C	110.3
C17—C18—H18A	109.5	C36B—C35B—H35C	110.3
C17—C18—H18B	109.5	O8—C35B—H35D	110.3
H18A—C18—H18B	109.5	C36B—C35B—H35D	110.3
C17—C18—H18C	109.5	H35C—C35B—H35D	108.5
H18A—C18—H18C	109.5	C35B—C36B—H36D	109.5
H18B—C18—H18C	109.5	C35B—C36B—H36E	109.5
N3—C19—C20	109.91 (9)	H36D—C36B—H36E	109.5
N3—C19—C24	110.78 (8)	C35B—C36B—H36F	109.5
C20—C19—C24	110.58 (8)	H36D—C36B—H36F	109.5
N3—C19—H19	108.5	H36E—C36B—H36F	109.5

### Hydrogen-bond geometry (Å, °)

**Table:**

*D*—H···*A*	*D*—H	H···*A*	*D*···*A*	*D*—H···*A*
N1—H1N···O1	0.850 (13)	2.048 (14)	2.754 (2)	140.1 (12)
N2—H2N···O3	0.865 (12)	2.009 (12)	2.720 (2)	138.7 (13)
N3—H3N···O5	0.846 (17)	2.018 (15)	2.724 (2)	140.3 (12)
N4—H4N···O7	0.811 (17)	2.112 (15)	2.754 (2)	136.1 (14)
C4—H4A···O6A^i^	0.97	2.48	3.400 (3)	158

Symmetry codes: (i) *x*+1, *y*+1, *z*.
